# Severe Fever with Thrombocytopenia Syndrome Virus Infection, Thailand, 2019–2020

**DOI:** 10.3201/eid2812.221183

**Published:** 2022-12

**Authors:** Patthaya Rattanakomol, Sarawut Khongwichit, Piyada Linsuwanon, Keun Hwa Lee, Sompong Vongpunsawad, Yong Poovorawan

**Affiliations:** Chulalongkorn University, Bangkok, Thailand (P. Rattanakomol, S. Khongwichit, S. Vongpunsawad, Y. Poovorawan);; US Army Medical Directorate–Armed Forces Research Institute of Medical Sciences, Bangkok, Thailand (P. Linsuwanon);; Hanyang University, Seoul, South Korea (K.H. Lee)

**Keywords:** severe fever with thrombocytopenia syndrome virus, SFTS, SFTSV, vector-borne infections, viruses, phleboviruses, febrile illnesses, infections, Thailand, Asia

## Abstract

Infection with severe fever with thrombocytopenia syndrome (SFTS) virus, which can cause hemorrhagic febrile illness, is often transmitted by ticks. We identified 3 patients with SFTS in or near Bangkok, Thailand. Our results underscore a need for heightened awareness by clinicians of possible SFTS virus, even in urban centers.

Severe fever with thrombocytopenia syndrome (SFTS) is a tickborne viral disease associated with acute fever, possibly accompanied by vomiting, diarrhea, fatigue, myalgia, and leukocytopenia (*1*). Most reports of infection have come from studies in South Korea, Japan, and China, although Taiwan, Vietnam, and Myanmar have had confirmed cases in recent years (*2*). Severe infections can cause hemorrhagic fever and multiple organ failure leading to death. SFTS results from infection by the SFTS virus (SFTSV, newly renamed *Dabie bandavirus*), an RNA virus in the family Phenuiviridae, genus *Bandavirus* (*3*). More frequent arbovirus infections in Thailand, primarily dengue and chikungunya, often confound diagnosis of febrile illness caused by other viruses such as SFTSV because most clinicians lack awareness. 

Testing during an upsurge in chikungunya virus infection in Thailand at the end of 2018 found that >70% of acute febrile illnesses were laboratory-confirmed chikungunya (*4*). As the proportion of chikungunya virus–positive samples eventually decreased, we began to screen for other common viral etiologies of acute fever, including dengue and Zika viruses (Appendix Figure). Because SFTSV had been reported in 2 patients in Vietnam (*5*) at the time, when the samples from Thailand tested negative for all 3 more common viruses, we began examining for possible SFTSV infection. The Institutional Review Board of Chulalongkorn University Faculty of Medicine approved this study (IRB number 0453/65). 

We subjected de-identified archived RNA samples from 712 patients from Bangkok and surrounding areas, hospitalized during October 2018–March 2021, to reverse transcription PCR to detect the nucleoprotein gene region of the small (S) segment of SFTSV (*6*). Three samples tested positive, so we used 3 primer sets described elsewhere (*7*) to determine full-length S-segment nucleotide sequences. We deposited sequences in the GenBank database (accession numbers ON840548–50) and constructed the SFTSV S-segment phylogenetic tree using MEGA11 (https://www.megasoftware.net).

Phylogenetic analysis suggested that the 3 SFTSV strains from Thailand shared ≈99.7% nucleotide sequence identities and were genetically closest to the SFTSV strains from China identified in 2012–2017 (99.3%–99.6% nucleotide identity) (Figure; Appendix Table). On the basis of available clinical records, all 3 patients reported myalgia with lower than normal leukocyte (<3,000 cells/μL) and platelet (<110,000 cells/μL) counts (Table). Two patients experienced elevated alanine and aspartate aminotransferase levels (>60 U/L). Although patient 1 did not demonstrate substantially altered leukocyte count or blood chemistry, he experienced gastrointestinal symptoms (abdominal pain, nausea, vomiting, and diarrhea). 

**Figure:**
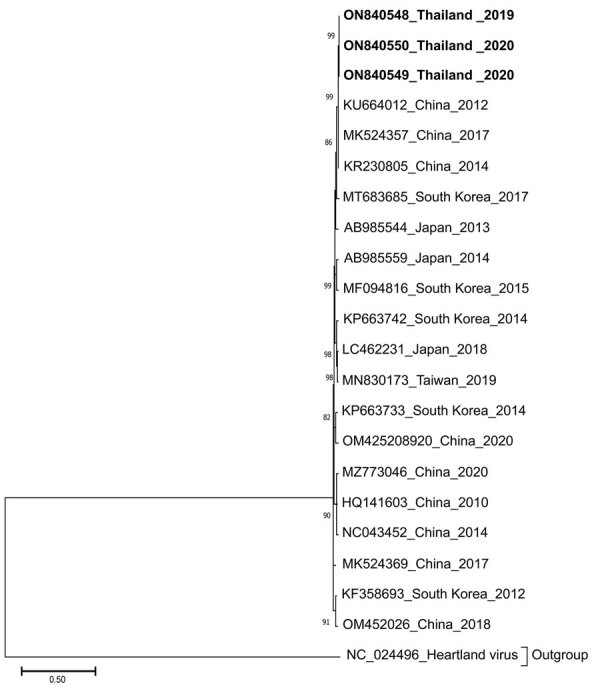
Phylogenetic analysis of the S segment sequence (1,674 bp) of 3 SFTSV strains from Thailand (bold) compared with reference sequences. The tree was generated using the maximum-likelihood method based on the Kimura 2-parameter model with 1,000 bootstrap replicates. Strains are noted with GenBank accession numbers, country, and year of isolation. Bootstrap values >70% are indicated at the branch nodes. Scale bar indicates the number of substitutions per site.

**Table:** Characteristics and detection of severe fever with thrombocytopenia syndrome virus in 3 patients in Thailand, 2019–2020.

Category	Patient 1	Patient 2	Patient 3
Age, y/sex	60/M	16/M	52/F
Location	Bangkok	Chachoengsao	Bangkok
Collection date	2019 Nov 14	2020 May 10	2020 Oct 19
Clinical manifestations			
Temperature	37.2°C	40.6°C	38.1°C
Clinical signs and symptoms	Myalgia, arthralgia, cough, nausea, vomiting, abdominal pain, diarrhea	Myalgia	Myalgia, arthralgia
Laboratory findings (reference range)			
Leukocytes, cells/µL (4,100–10,900)	1,790	900	2,770
Neutrophils, % (40–72)	45	31	62
Lymphocytes, % (18–49)	42	59	34
Platelets, cells/µL (140,000–400,000)	107,000	45,000	121,000
Aspartate aminotransferase, U/L (<40)	Not done	102	1,758
Alanine aminotransferase, U/L (<41)	24	63	973
Pathogens tested for but not detected	*Rickettsia/Orientia* spp., influenza A/B	*Rickettsia/Orientia* spp., influenza A/B, Epstein-Barr, hepatitis B/C, SARS-CoV-2, malaria, *Leptospira*, *Burkholderia pseudomallei*	*Rickettsia/Orientia* spp., hepatitis A/B

Patients reported no history of travel within 3 weeks before symptom onset. Patients 1 and 3 lived in Bangkok, whereas patient 2 lived in Chachoengsao Province, ≈40 km east of Bangkok. Because international travel was severely limited during the global coronavirus pandemic beginning in 2020, transboundary transmission of SFTSV was unlikely.

A study in South Korea found that roughly one quarter of SFTSV infections accompanied scrub typhus caused by *Orientia tsutsugamushi* infection (*8*) and suggested the possibility of the chigger mite as a potential vector of the virus. To further investigate potential co-infection with tickborne and chiggerborne bacteria, we performed multiplexed quantitative PCR to detect *Orientia* and *Rickettsiae* spp. None of our 3 patients tested positive for these bacteria.

Our study identified SFTSV by analyzing febrile illnesses among patients who previously tested negative for arboviruses typically suspected of causing acute fever in Thailand. We found that <0.5% of these samples tested positive for SFTSV, which represented only 0.1% prevalence when all febrile illnesses were considered. However, this percentage might be higher in patients residing in rural areas or who engage in agriculture. A strength of this study was confirmation of SFTSV from full-length S-segment nucleotide sequences from 3 symptomatic patients residing in urban areas during November 2019–October 2020. 

We do not know how the patients in our study contracted SFTSV, particularly the identities of any likely reservoir hosts and arthropod vectors, because of limited available clinical information regarding viral exposure. To date, 4 tick species are known vectors for SFTSV: *Haemaphysalis longicornis*, *Amblyomma testudinarium*, *Rhipicephalus microplus*, and *Ixodes nipponensis*, the last of which is not present in Thailand (*9**,**10*). *R. microplus* is often found in livestock animals in countries in Southeast Asia, but all 3 patients in our study were urban dwellers. Our study was limited by using data from retrospective evaluation of clinical records, which could have been more comprehensive had physicians initially suspected SFTS. Also, the fact that patients did not travel internationally could not rule out domestic rural exposure to SFTSV. Another limitation was that insufficient S-segment sequences from SFTSV strains previously identified in countries in Southeast Asia, such as Myanmar and Vietnam, were available in the public database, preventing direct genetic comparison with the strains from Thailand identified in this study. Nevertheless, our finding of detailed molecular evidence of SFTSV infection in Thailand, although in very few patients to date, should increase awareness of SFTS and warrants further exploration into possible transmission cycles in tropical urban settings.

Appendix Addition information about study of severe fever with thrombocytopenia syndrome in Thailand.
