# Supplementary material for: Severe Fever with Thrombocytopenia Syndrome Virus Infection, Thailand, 2019–2020
Source: Emerg Infect Dis. 2022 Dec;28(12):2572–4. doi: 10.3201/eid2812.221183 (PMC9707585; doi:10.3201/eid2812.221183)
Supplement: Appendix — Addition information about study of severe fever with thrombocytopenia syndrome in Thailand. [file 22-1183-Techapp-s1.pdf]

Article DOI: https://doi.org/10.3201/eid2812.221183

Severe Fever with Thrombocytopenia Syndrome Virus Infection, Thailand, 2019–2020

Appendix

Appendix Table. Comparison of the nucleotide identities of the S (small) segment sequence among the SFTSV strains*

No.	SFTSVS segment sequence	1	2	3	4	5	6	7	8	9	10	11	12	13	14	15	16	17	18	19	20	21
1	ON840548_Thailand_2019	ID	99.8	99.8	99.2	99.4	99.2	96.5	96.0	95.6	95.6	95.6	95.8	95.4	94.5	94.5	94.5	94.9	94.4	94.8	94.3	94.3
2	ON840549_Thailand_2020	99.8	ID	99.8	99.1	99.3	99.1	96.5	96.1	95.7	95.7	95.7	95.9	95.5	94.5	94.5	94.7	94.9	94.4	94.8	94.3	94.4
3	ON840550_Thailand_2020	99.8	99.8	ID	99.1	99.3	99.1	96.5	95.9	95.7	95.7	95.7	95.9	95.5	94.5	94.4	94.5	94.8	94.3	94.7	94.4	94.2
4	KU664012_China/2012	99.2	99.1	99.1	ID	99.8	99.5	96.8	96.3	95.9	95.9	96.1	96.1	95.7	94.8	94.8	94.7	95.0	94.6	95.1	94.7	94.6
5	MK524357_China/2017	99.4	99.3	99.3	99.8	ID	99.7	97.0	96.5	96.1	96.1	96.1	96.3	95.9	95.0	95.0	94.9	95.2	94.8	95.3	94.9	94.8
6	KR230805_China_2014	99.2	99.1	99.1	99.5	99.7	ID	97.1	96.6	96.2	96.2	96.1	96.4	95.9	95.2	95.0	95.0	95.3	94.9	95.2	94.9	94.8
7	MT683685_South Korea_2017	96.5	96.5	96.5	96.8	97.0	97.1	ID	96.2	95.7	95.9	95.6	95.7	95.4	94.4	94.6	94.6	94.5	94.5	95.1	94.7	94.6
8	AB985544_Japan_2013	96.0	96.1	95.9	96.3	96.5	96.6	96.2	ID	96.3	96.6	95.9	96.1	95.8	95.3	95.1	94.9	95.2	94.9	95.2	94.8	94.9
9	AB985559_Japan_2014	95.6	95.7	95.7	95.9	96.1	96.2	95.7	96.3	ID	98.0	95.9	96.3	95.9	94.7	94.7	94.9	95.2	94.8	95.4	94.8	94.8
10	MF094816_South Korea_2015	95.6	95.7	95.7	95.9	96.1	96.2	95.9	96.6	98.0	ID	96.4	96.6	96.3	94.9	94.8	95.2	95.6	95.1	95.4	95.1	94.9
11	KP663742_South Korea_2014	95.6	95.7	95.7	96.1	96.1	96.1	95.6	95.9	95.9	96.4	ID	97.9	97.8	95.4	95.3	95.1	95.1	94.8	95.8	95.2	95.0
12	LC462231_Japan_2018	95.8	95.9	95.9	96.1	96.3	96.4	95.7	96.1	96.3	96.6	97.9	ID	98.6	95.2	95.3	94.9	95.1	94.8	95.5	95.1	94.9
13	MN830173_Taiwan_2019	95.4	95.5	95.5	95.7	95.9	95.9	95.4	95.8	95.9	96.3	97.8	98.6	ID	95.1	95.1	94.8	95.0	94.7	95.3	94.9	94.7
14	KP663733_South Korea_2014	94.5	94.5	94.5	94.8	95.0	95.2	94.4	95.3	94.7	94.9	95.4	95.2	95.1	ID	97.4	95.1	95.3	95.1	95.6	95.0	94.8
15	OM45208920_China_2020	94.5	94.5	94.4	94.8	95.0	95.0	94.6	95.1	94.7	94.8	95.3	95.3	95.1	97.4	ID	95.1	95.2	94.9	95.9	94.7	94.5
16	MZ773046_China_2020	94.5	94.7	94.5	94.7	94.9	95.0	94.6	94.9	94.9	95.2	95.1	94.9	94.8	95.1	95.1	ID	98.2	98.0	95.4	94.6	94.7
17	HQ141603_China_2010	94.9	94.9	94.8	95.0	95.2	95.3	94.5	95.2	95.2	95.6	95.1	95.1	95.0	95.3	95.2	98.2	ID	98.5	95.4	95.2	95.1
18	NC043452_China_2014	94.4	94.4	94.3	94.6	94.8	94.9	94.5	94.9	94.8	95.1	94.8	94.8	94.7	95.1	94.9	98.0	98.5	ID	95.3	95.0	94.8
19	MK524369_China_2017	94.8	94.8	94.7	95.1	95.3	95.2	95.1	95.2	95.4	95.4	95.8	95.5	95.3	95.6	95.9	95.4	95.4	95.3	ID	96.6	96.3
20	KF358693_South Korea_2012	94.3	94.3	94.4	94.7	94.9	94.9	94.7	94.8	94.8	95.1	95.2	95.1	94.9	95.0	94.7	94.6	95.2	95.0	96.6	ID	98.3
21	OM452026_China_2018	94.3	94.4	94.2	94.6	94.8	94.8	94.6	94.9	94.8	94.9	95.0	94.9	94.7	94.8	94.5	94.7	95.1	94.8	96.3	98.3	ID

*SFTSV, severe fever with thrombocytopenia syndrome virus; ID, identity percentage not applicable

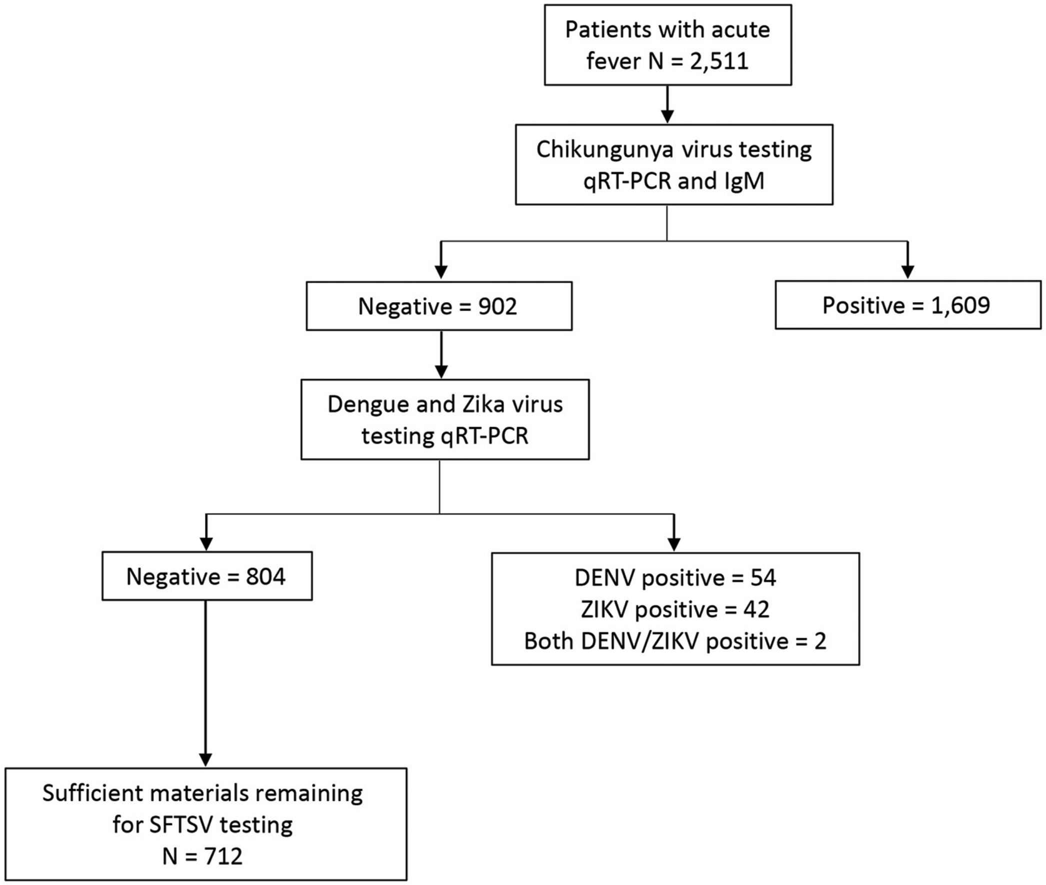

Appendix Figure. Febrile illness samples chosen for SFTSV testing
